# 38.8 million additional modern contraceptive users: this, in fact, *is* “a never-before opportunity to strengthen investment and action on adolescent contraception”

**DOI:** 10.1186/s12978-018-0457-z

**Published:** 2018-01-30

**Authors:** Venkatraman Chandra-Mouli, Marina Plesons, Emily Sullivan, Lianne Gonsalves, Lale Say

**Affiliations:** 1 World Health Organization, Geneva, Switzerland; 2 Family Planning 2020, Washington, USA

**Keywords:** Adolescent health, Adolescent pregnancy, Contraceptive availability, Contraceptive distribution, Adolescent health services, Sustainable development goals, Family planning, Sexual health, Reproductive health

## Abstract

**Background:**

We thank Bijlmakers et al. for their interest in our article, “A never-before opportunity to strengthen investment and action on adolescent contraception, and what we must do to make full use of it”, and are grateful for the opportunity to respond to their four key assertions.

**Response:**

First, we fully agree that sexual rights are controversial, which we discussed in depth in our original article. However, we reaffirm that there is global consensus on adolescent contraception as evidenced in part by recent data emerging from FP2020 on 38.8 million additional modern contraceptive users, the Global Goods and commitments emanating from the 2017 FP2020 summit, and their translated actions at the country level. Additionally, we clarify WHO’s working definitions of sex, sexual health, and sexuality, and introduce WHO’s newly released Operational Framework on Sexual Health and its Linkages to Reproductive Health. We welcome and agree with Bijlmakers et al.’s second point, which elaborates on the barrier of restrictive laws and policies. To address this barrier, we describe examples of resources that can help programmes understand the political/social context that drives these laws and policies at national and subnational levels, and identify programmatic gaps and best practices to address them within specific political/social contexts. We also welcome and agree with Bijlmakers et al.’s third point, which reiterates that discomfort around adolescent sexuality is a major barrier for sexuality education. In response, we point to four relevant reviews of CSE policies and their implementation, our original article’s description of three programmes that have successfully addressed inadequate teacher skills, and our ongoing work on documenting strategies to build an enabling environment for CSE and deal with resistance. Lastly, we wholeheartedly agree that the harmful policies noted by Bijlmakers et al. are damaging to international efforts to improve adolescent SRH and rights. We argue, though, that these policies alone will not undermine efforts by countless other stakeholders around the world who are working in defence and promotion of adolescents’ SRH and rights.

**Conclusion:**

Despite the many valid obstacles noted by Bijlmakers et al., we truly believe that this is “a never-before opportunity to strengthen investment and action on adolescent contraception”.

## Background

We thank Bijlmakers et al. [1] for their interest in our article, “A never-before opportunity to strengthen investment and action on adolescent contraception, and what we must do to make full use of it” [2]. Our commentary was intended to stimulate discourse, and we thus welcome their engagement. We are pleased to have the opportunity to continue this discussion by responding to Bijlmakers et al.’s four key points, which:

1. Point out that “sexual rights are controversial” [1] and question whether there is, in fact, real global consensus on adolescent contraceptive use;
2. Affirm that “political factors at the national level” [1] obstruct adolescent access to and use of contraception;
3. Elaborate on the need to address “ambivalence” to allow “trained teachers, civil society leaders and adult role models…to actually deliver evidence-based and positive messages about contraception” [1];
4. And argue that “one cannot remain silent” about “barriers at the global level” – namely “non-adherence to international commitments” through “promotion of abstinence-only-until-marriage (AOUM) policies” and the “re-enactment and expansion of the Mexico City Policy, widely known as the Global Gag Rule” [1].

### A second look at the global consensus on adolescent contraceptive use

Bijlmakers et al. question whether there is, in fact, real global consensus on adolescent contraceptive use. They point out that “sexual rights are contested” and that “in many societies there is a general fear and anxiety surrounding adolescent sexuality” [1]. We are in complete agreement that discomfort around adolescent sexuality and sexual rights is common and that there is resistance to providing contraceptives to adolescents, especially to those who are unmarried. We discuss these issues in the section entitled “Why are adolescents still unable to obtain and use contraceptives?” [2]. We note barriers to adolescents’ access to contraceptives at various levels of the ecological framework: restrictive laws and policies regarding provision of contraception based on age or marital status; health worker bias and/or lack of willingness to acknowledge adolescents’ sexual health needs; and adolescents’ own inability to access contraceptives because of knowledge, transportation, and financial constraints. Additionally, we describe barriers that prevent use/consistent use of contraception, even when adolescents are able to obtain contraceptives: pressure to have children; stigma surrounding non-marital sexual activity and/or contraceptive use; fear of side effects; lack of knowledge on correct use; and factors contributing to discontinuation (i.e. hesitation to go back and seek contraceptives because of negative first experiences with health workers/health systems, changing reproductive needs, changing reproductive intentions).

Although there is not universal support for adolescent contraception and sexual rights, we stand by our original assertion that this is a never-before opportunity to address adolescent contraception. As compared to 2012, 38.8 million additional women and girls are using modern contraception [3]. At this year’s FP2020 summit, three Global Goods were announced to specifically strengthen the family planning sector’s ability to meet the needs of young people: the Youth Accountability Framework, the Global Adolescent Data Statement, and the Partnership to Strengthen Country Capacity [3]. Additionally, 41 partner countries, 14 donor countries, 39 civil society partners, 9 foundations, 4 multilaterals, and18 private sector partners agreed to a declaration which included the following message, “As the generation of the future, it is our collective responsibility to empower [adolescents] to thrive, and doing so is central to achieving the FP2020 & broader Sustainable Development Goals…” [4]. In comparison to the 2012 Summit, more countries (35 in total) made additional commitments to specific and evidence-based financial, political, and programmatic actions focused on adolescents and youth, including providing free contraceptives to adolescents, scaling-up youth-friendly services, and implementing comprehensive sexuality education (CSE) programs for those in- and-out of school [4].

These commitments are not token statements. Building off their FP2020 commitments and corresponding Global Financing Facility investment cases, Mozambique and Liberia, for example, are expanding their prioritization of adolescent contraception in prominent ways. Mozambique has initiated provision of sexual and reproductive health (SRH) services, including contraceptives, in secondary schools and is strengthening referral arrangements between school-based health facilities and nearby public/private health facilities, aiming for national coverage by 2020 [5]. Liberia is scaling up youth-friendly health services, including free-of-charge provision of contraceptives, in health facilities and is implementing “complementary approaches in community outreach, social marketing, and commercial sales” [6, 7].

**Fig. 1:**
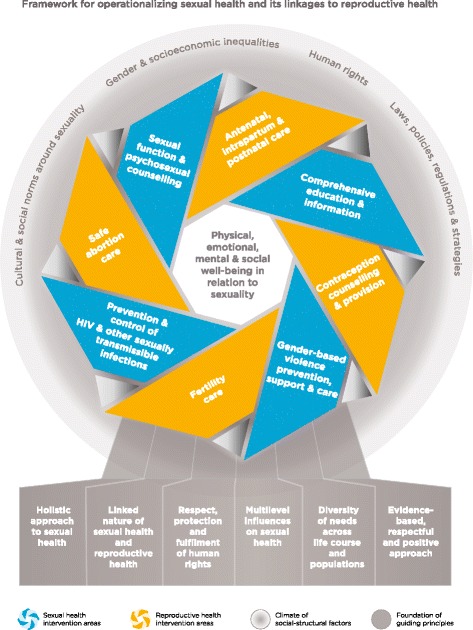
WHO Framework for operationalizing sexual health and its linkages to reproductive health

Bijlmakers et al. also assert that the Human Reproduction Programme (HRP), on behalf of the United Nations, “provides definitions of sex, sexual health and sexuality. However for sexual rights…the webpage merely provides a working definition” [1]. All four definitions are, in fact, working definitions that were developed in a 2002 technical consultation on sexual health [8], with the definition of sexual rights, in particular, being enhanced in a 2010 document [9]. Sexual rights, sex, sexuality, and sexual health have been, and continue to be, concepts addressed in the HRP’s work. Most recently, the WHO released an *Operational Framework on Sexual Health and its Linkages to Reproductive Health* (Fig. 1), developed precisely because “in certain settings and for certain populations, crucial aspects of [sexual health] may be overlooked when sexual health is grouped under or together with the domain of reproductive health” [10]. The framework reinforces that comprehensive SRH services must comprise both sexual health *and* reproductive health interventions in order to be responsive to the needs of all individuals, with interventions then able to be tailored to particular populations in response to specific needs that may arise at certain points in the life course and in response to various circumstances.

### An elaboration of barriers: Restrictive laws and policies

We welcome and agree with Bijlmakers et al.’s second point, which reiterates that “national laws and policies vary a great deal” [1] in their support for adolescents’ access to contraception. We believe this message echoes and supports assertions conveyed in our commentary, and we appreciate their expanded discussion.

Bijlmakers et al. “endorse [our] call for implementation research that sheds light on context-specific programmatic challenges and employs methods to overcome identified obstacles” [1]. To change restrictive laws and policies, it is essential to first understand the political and social context that drives them at the national level and, in countries with decentralized political structures, at sub-national levels. Both WHO and Population Reference Bureau offer analytic frameworks for assessing these factors [11, 12]. Similarly, comprehensive programmatic reviews and situation analyses, such as those conducted by the Evidence Project in Bangladesh, are useful for identifying best practices and programmatic gaps within specific political, social, and programmatic contexts [13].

### A second elaboration of barriers: Discomfort about sexuality education

We also welcome Bijlmakers et al.’s third point, which reiterates that “trained teachers, civil society leaders, and adult role models” face difficulties in delivering “evidence-based and positive messages about contraception” [1]. We absolutely agree that there continues to be an enormous amount of discomfort about CSE, despite recognition of the need for CSE; growing acceptance that CSE is a right; availability of evidence on effectiveness and cost effectiveness, along with tools to advocate, plan, monitor, and evaluate programs; and inclusion of CSE in international declarations and regional/national plans of action. Programmes continue to manifest weak content (i.e. curricula include inadequate information about contraception and key aspects of SRH) and weak delivery (i.e. teachers lack trainings, skills, and comfort). Reviews by the Guttmacher Institute of CSE policies and their implementation in Ghana, Guatemala, Peru, and Kenya found that teachers face major challenges in delivering CSE, including those related to time, resources, and comfort [14–17].

Bijlmakers et al. rightfully state that “new avenues need to be explored that allow for accurate and positive teaching of adolescents about contraception in socio-cultural and political environments that are ambivalent about adolescent sexuality” [1]. In our commentary, we note that addressing “inadequate teacher skills”, alongside a number of other programmatic challenges, has “great potential to increase the effectiveness of CSE programmes”, and we describe a few examples of programmes that have done so (i.e. Geracao Biz in Mozambique, Udaan in India, and the national school-based CSE programme in Estonia) [2]. Furthermore, in order to understand discomfort at various levels, WHO is documenting a series of case studies of strategies that programmes have successfully used to build an enabling environment for CSE and deal with resistance [18, 19].

### An acknowledgment of harmful policies, but a focus on efforts to minimize their negative effects

Bijlmakers et al. assert that “one cannot remain silent” about “barriers at the global level” – namely “non-adherence to international commitments” through “promotion of…AOUM policies” and the “re-enactment and expansion of the Mexico City Policy, widely known as the Global Gag Rule” [1].

We wholeheartedly agree that these policies are damaging to international efforts to improve adolescent SRH and rights. In February 2017, HRP’s Scientific and Technical Advisory Group officially declared, “We…are deeply concerned that current global trends will restrict access to life-saving SRH services and information for women and girls, especially those most in need, and fear that the significant gains made over the past three decades will be compromised [20]. In this context, WHO has consistently called for the provision of CSE in its own guidelines, based on extensive evidence that AOUM curricula are not effective [21]. Along with other UN agencies, WHO also contributed to UNESCO’s International Technical Guidelines on Sexuality Education in 2009, along with the forthcoming 2018 updated revision that encourages discussion of sexuality in a positive light” [21, 22].

Bijlmakers et al. are correct in their assertion about the trend in “putting ideology before evidence” [1], and it is necessary to be aware of such resistance; however, it cannot and will not stop efforts to realize SRH and rights around the world. WHO has continued – most recently, in the context of the Sustainable Development Goals (with 3.7 in particular calling for universal access to SRH services) – to encourage that the SRH needs of all populations, at all ages and in all settings, be acknowledged and incorporated into evidence-based research and programming.

Furthermore, as Bijlmakers et al. themselves note, these global trends have sparked renewed commitments from a wide range of stakeholders that demonstrate “heartening efforts to boost support for women’s SRH and rights” [1]. Bijlmakers et al. describes the ‘She Decides’ movement led by the Dutch Minister of Foreign Trade and Development Cooperation, which has been “endorsed by more than 30 governments” [1]. To a similar aim, Canada has initiated a Feminist International Assistance Policy, which will specifically invest $650 M over 3 years to “[increase] access to a full range of health services, including family planning and modern contraception; CSE; safe and legal abortion, and post-abortion care” [23]. We view these as perfect examples that reaffirm the global consensus on adolescent contraceptive use, and SRH and rights more broadly. While we agree that AOUM and the Mexico City Policy are “problematic” [1] and that they cause problems with contraceptive service provision, they alone will not undermine efforts by countless other stakeholders around the world who are working in defence and promotion of adolescents’ SRH and rights.

## Conclusion

Despite the many valid obstacles noted by Bijlmakers et al., 38.8 million additional women and girls are using modern contraception compared to 2012 [3]. This *is*, in fact, “a never-before opportunity to strengthen investment and action on adolescent contraception” [2].

## Abbreviations

AOUM: Abstinence-only-until-marriage
CSE: Comprehensive sexuality education
FP2020: Family Planning 2020
HRP: Human Reproduction Programme
SRH: Sexual and reproductive health
UNESCO: United Nations Educational, Scientific, and Cultural Organization
WHO: World Health Organization
